# Precision Medicine in Overlap Syndrome (COPD–Obstructive Sleep Apnea): From Phenotypes and Endotypes to Treatable Traits

**DOI:** 10.3390/arm94050063

**Published:** 2026-09-01

**Authors:** Carina Adina Afloarei, Andreea Zabara Antal, David Toma, Adriana Loredana Pintilie, Georgiana Pitusac, Raluca Tiron, Tudor Birladeanu, Teodor Zaharia, Radu Crisan Dabija

**Affiliations:** 1 Clinical Hospital of Pulmonary Diseases Iasi, 700116 Iasi, Romania; carina_adina98@yahoo.com (C.A.A.); andreea.zabara@pneumo-iasi.ro (A.Z.A.); prisacaru.georgiana@yahoo.com (G.P.); tironraluca@yahoo.com (R.T.); birladeanu.tudor@yahoo.com (T.B.); teozaharia96@gmail.com (T.Z.); radu.dabija@umfiasi.ro (R.C.D.); 2 Grigore T. Popa University of Medicine and Pharmacy, 700116 Iasi, Romania; pintilie.adriana.loredana.mg6.7@gmail.com

**Keywords:** COPD-OSA overlap syndrome, precision medicine, endotype, phenotype, biomarker, dupilumab, incretin-based therapies, double-hit hypoxemia

## Abstract

**Highlights:**

**What are the main findings?**

- COPD-OSA overlap syndrome is clinically and biologically heterogeneous, supporting the identification of distinct clinical phenotypes and molecular endotypes.
- A conceptual framework comprising four clinical phenotypes and three molecular endotypes highlights distinct pathophysiological mechanisms and potentially treatable traits.

**What is the implication on the main finding?**

- Endotype oriented assessment may support a more individualized approach to PAP therapy and targeted treatment in selected patients with OVS.
- Accessible biomarkers may help identify the predominant biological profile and provide a practical starting point for precision medicine in OVS.

**Abstract:**

Introduction: COPD-OSA overlap syndrome (OVS) is defined by the coexistence of chronic obstructive pulmonary disease and obstructive sleep apnea syndrome in the same patient; it affects 28.3% of patients evaluated for either condition and is associated with significantly higher mortality compared to either pathology in isolation. Current therapeutic approaches, involving PAP therapy and bronchodilation, treat this syndrome as a homogeneous entity and ignore the biological heterogeneity of this patient population. Objectives: This review proposes a systematic framework for the endotypic classification of biological interactions between COPD and OSA, integrating current literature on the pathophysiological mechanisms underlying the OVS, clinically accessible biomarkers, and emerging therapies. Methods: A narrative review based on available literature, focusing on studies published between 2010 and 2026 identified via searches in PubMed, PMC, and Dove Medical Press using the terms: COPD-OSA overlap syndrome, endotype, precision medicine, phenotype, biomarker, dupilumab, and incretin-based therapies. Results: Four clinical phenotypes (obese-metabolic, emphysematous, bronchitic-hypoxemic, and hypercapnic) and three molecular endotypes (Th2/eosinophilic, neutrophilic/oxidative, and metabolic-adipokine) are proposed, each with distinct pathophysiological mechanisms and specific therapeutic implications. The interaction between the two conditions generates a unique, pronounced hypoxemic profile. We propose the “double-hit hypoxemia” model as a conceptual framework characterized by amplified systemic inflammation and increased cardiovascular risk compared to either pathology in isolation. This proposed model has not yet been prospectively validated. Dupilumab (approved for an inflammatory phenotype in COPD patients characterized by eosinophil counts ≥ 300 cells/μL) represents a promising option, though currently unsupported in the OVS population, that could nonetheless be relevant to the Th2/eosinophilic endotype of this syndrome and tirzepatide (which reduced the AHI by up to 23.8 events/hour versus placebo in the SURMOUNT-OSA trial, conducted in patients with obesity and moderate-to-severe OSA, rather than in patients with confirmed OVS) could represent a promising therapy for the metabolic-adipokine endotype of obese patients with OVS. A minimal biomarker panel, comprising blood eosinophils, FeNO, daytime PaCO_2_, BMI, and T90, allows for a practical approach to identifying the dominant endotype. Conclusions: Endotyping of OVS may provide a framework for moving beyond a uniform therapeutic approach toward more individualized management based on the dominant underlying biological mechanism. Randomized clinical trials focusing on endotypic stratification and OVS cohorts remain research priorities.

## 1. Introduction

Chronic obstructive pulmonary disease (COPD) and obstructive sleep apnea (OSA) are pulmonary conditions frequently encountered in medical practice. COPD is a heterogeneous condition characterized by chronic respiratory symptoms (dyspnea, cough, sputum production, exacerbations) resulting from persistent airway and/or alveolar abnormalities that typically lead to persistent and progressive airflow obstruction [1]. It poses a challenge to global healthcare systems, carrying significant socioeconomic consequences [2]. In 2020, the global prevalence was 10.6% (equivalent to 480 million cases) and the number of cases is expected to increase by 23% until 2050 [3]. Obstructive Sleep Apnea is a condition characterized by repeated episodes of complete (apnea) or partial (hypopnea) upper airway collapse, leading to a drop in oxygen saturation or patient arousal from sleep [4]. These recurrent episodes alter sleep architecture, resulting in a non-restorative and fragmented sleep pattern. Other highly suggestive symptoms include snoring, witnessed apneas, and excessive daytime sleepiness [5,6,7]. OSA significantly increases cardiovascular risk, morbidity, and mortality, particularly in the context of the hypoxia already present in COPD. This coexistence was first documented by Flenley in 1985 and is far more common than previously estimated.

A meta-analysis of 41 studies involving 1.8 million participants estimated the prevalence of this coexistence at 28.3% (95% CI: 20.8–36.4%) among those evaluated for either condition, with prevalence rising substantially between 2015 and 2025, in parallel with the global obesity pandemic [8]. Overlap syndrome (OVS) is not merely a combination of these two conditions; a study by Marin et al. demonstrates that untreated OVS patients have a significantly worse prognosis compared to those with either condition alone: all-cause mortality was significantly higher in patients with untreated OVS compared with patients with COPD alone, with cardiovascular disease representing the leading cause of death. Effective CPAP therapy was associated with improved survival and a significant reduction in COPD exacerbations requiring hospitalization [9]. Despite four decades of clinical recognition and robust epidemiological evidence, OVS remains consistently underdiagnosed and undertreated, a paradox highlighted in a recent American Thoracic Society editorial, which notes that the two communities managing this patient population continue to operate largely in separate silos [10].

OVS continues to be approached therapeutically as a heterogeneous entity, with continuous positive airway pressure (CPAP) and bronchodilation serving as the pillars of patient care regardless of underlying pathophysiological mechanisms; however, this “one-size-fits-all” approach is becoming difficult to justify given the condition’s well-documented heterogeneity, ranging from non-obese patients with emphysema-predominant disease, mild OSA, and preserved gas exchange to those with morbid obesity, hypoxemia, and hypercapnia, a profile prone to developing pulmonary hypertension [11,12,13]. This incomplete approach stands in contrast to the progress made in isolated COPD, where precision medicine, driven by the identification of phenotypes, endotypes, biomarkers, and treatable traits, has transformed management over the last decade. Precision approaches targeting T2/eosinophilic inflammation have evolved. The literature indicates a reduction in moderate-to-severe exacerbations among COPD patients treated with the anti-IL-5 agent mepolizumab as an add-on to triple therapy in the presence of elevated eosinophil counts (MATINEE Phase 3 trial), thereby supporting the initiation of such therapy in these populations [14]. These data support a biomarker-guided approach to the use of both inhaled corticosteroids (ICS) and biologic therapies in patients with an eosinophil-predominant endotype (minimal effect at <100 cells/µL; greatest effect at ≥300 cells/µL) [15]. The literature lacks high-quality interventional studies addressing OVS phenotypes; therapeutic insights are derived from patient subgroups, as guidelines do not address individual clinical scenarios [11]. This conceptual gap, namely the application of precision medicine to OVS, defines both the current practical limitation and the central objective of this review. Previous reviews of OVS have highlighted epidemiological data, diagnostic criteria, and general therapeutic management. The present paper aims to propose an integrated framework that combines the classification of phenotypes and endotypes with an accessible biomarker panel and the most recent data on targeted therapies, such as biologic therapy for type 2 inflammation and incretin-based therapies for metabolic dysfunction.

This review therefore moves beyond the traditional description of OVS and provides a systematic framework for the endotypic classification of molecular interactions between COPD and OSA. Drawing on existing literature, we propose three main molecular endotypes (eosinophilic-inflammatory, neutrophilic/oxidative, and metabolic-adipokine), each characterized by distinct pathophysiological mechanisms, accessible clinical biomarkers, and therapeutic implications. We also aim to incorporate recent data on emerging therapies, specifically GLP-1 receptor agonists (the SURMOUNT-OSA trial) and dupilumab (the BOREAS and NOTUS trials), which underscore the need for targeted therapy based on the dominant endotype, demonstrating that these treatments are not universally applicable to all patients but rather to specific profiles.

## 2. Methods

This is a narrative review of the literature published between 2010 and 2026, based on searches conducted in PubMed in July 2026, using the terms: COPD-OSA overlap syndrome, endotype, precision medicine, phenotype, biomarker, dupilumab, and GLP-1 receptor agonists. PMC was used for full-text access, and Dove Medical Press was additionally searched for relevant open-access publications. Formal PRISMA methodology was not applied, given the narrative nature of this review. Articles in English addressing the pathophysiology, biomarkers, phenotypes/endotypes, or management of COPD-OSA overlap syndrome were included; case reports and conference abstracts without a corresponding full-text publication were excluded. Given the narrative nature of the review, the total number of articles identified, screened, and excluded was not recorded, and the assessment of quality and risk bias was not performed. A total of 102 references were included. Embase, Scopus, and Web of Science were not searched, which is acknowledged as a limitation.

## 3. A Proposed ‘Double-Hit’ Hypoxemia Model

Based on the available literature and the described pathophysiological mechanisms, we propose a conceptual model of hypoxemia in OVS, which we term the “double-hit” model. This model represents our synthesis of the existing evidence and not an established or independently validated model. The relevance and applicability of this proposed model require confirmation through prospective studies conducted on well-characterized cohorts of patients with OVS.

### 3.1. Mechanical Interactions Between the Upper Airways and the Lungs

Mechanical interactions represent a significant mechanism through which COPD influences susceptibility to sleep apnea syndrome. On one hand, chronic upper airway inflammation, ubiquitous in COPD, causes significant mucosal edema, as well as adenotonsillar hypertrophy and increased nasopharyngeal resistance; this promotes pharyngeal collapse during sleep, particularly in active smokers [16]. Evidence suggests that the use of nasal anti-inflammatory therapy (corticosteroids/montelukast) can lower resistance by reducing inflammation, thereby contributing to a decrease in the number of breathing pauses during sleep [17,18]. Pulmonary hyperinflation, a characteristic of COPD, especially in the emphysematous phenotype, exerts a caudal traction force on the trachea; this phenomenon stiffens the pharyngeal wall and reduces the likelihood of upper airway collapse during sleep, a mechanism known as “tracheal tug” [19]. Biselli et al. [20] demonstrated this, finding that the degree of hyperinflation observed on imaging correlated with lower OSA severity. These results argue against the hypothesis that the destruction of lung parenchyma and loss of elastic recoil associated with severe COPD would promote OSA. Instead, they raise the hypothesis that hyperinflation exerts a protective effect by maintaining this tracheal traction.

However, mechanical protection varies across phenotypes: the low BMI associated with the emphysematous phenotype enhances upper airway stability [21,22], whereas the bronchitic phenotype retains heightened susceptibility due to persistent inflammation and parapharyngeal adipose deposition [23] (phenotype-specific details are provided in Section 3). Furthermore, the generalized muscle dysfunction associated with COPD impairs pharyngeal dilator activity, thereby reducing the compensatory capacity to counteract negative pressure during sleep.

### 3.2. Synergistic Hypoxemia

Physiologically, sleep is associated with reduced transmission of neural impulses from respiratory centers to the brainstem [24]. This results in a generalized decrease in respiratory drive, the cessation of accessory respiratory muscle activity (with the exception of the diaphragm), and reduced tone in the upper airway dilator muscles, all of which predispose the airway to pharyngeal collapse [25]. These conditions alter arterial oxygen tension and increase carbon dioxide levels, shifts that cause respiratory control to rely increasingly on peripheral and central chemoreceptors [26,27]. In isolated OSA, desaturations are episodic and linked to the reopening of the upper airway. In isolated COPD, hypoxemia is persistent, chronic, and baseline in nature, becoming particularly exacerbated during REM sleep as a result of hypoventilation. The pathophysiological consequence of this coexistence is thought to be a synergistic interaction: the impaired gas exchange associated with COPD limits compensatory responses to OSA-induced hypoxic events, leading over time to more severe and prolonged desaturations, while the reduced chemosensitivity seen in OSA combines with the intrinsically diminished ventilatory drive of COPD, resulting in further suppression [28,29,30,31]. A clinically significant point is that the hypoxemia or hypercapnia associated with OSA is disproportionate to the degree of bronchial obstruction observed via FEV1 measurements; consequently, blood gas analysis is essential in the management of these patients, regardless of the severity indicated by spirometry.

Hypoxia is a pathophysiological event present continuously in COPD and intermittently in OSA; it contributes to the development of a complex systemic inflammatory response through the release of systemic pro-inflammatory mediators such as CRP, IL-6, IL-8, TNF-α, and nuclear factor kappa-B (NF-κB) [32,33]. Furthermore, the intermittent phenomena associated with apnea episodes trigger oxidative stress via the generation of reactive oxygen species (ROS), predominantly within leukocytes, leading to endothelial dysfunction and the acceleration of the atherosclerotic process. The cumulative effect of these repetitive or persistent phenomena results in a significantly more severe cardiovascular and inflammatory profile compared to either of the two conditions considered in isolation [34].

### 3.3. Cardiovascular and Autonomic Amplification

Sympathetic nervous system activation occurs through different mechanisms. In OSA, repetitive micro-arousals and intermittent hypoxia directly activate the sympathetic nervous system, altering blood pressure and heart rhythm profiles; conversely, in COPD, hypoxemia and hypercapnia maintain elevated basal sympathetic tone with persistent sympathetic activation, even during wakefulness. Both conditions are independently recognized as cardiovascular risk factors through mechanisms involving endothelial dysfunction, systemic inflammation, and vascular remodeling. In OVS, the coexistence of these two pathologies creates a cumulative effect on these mechanisms, contributing to a higher risk of pulmonary hypertension, right heart failure, and atrial fibrillation compared to either disease alone [35,36].

**Figure 1:**
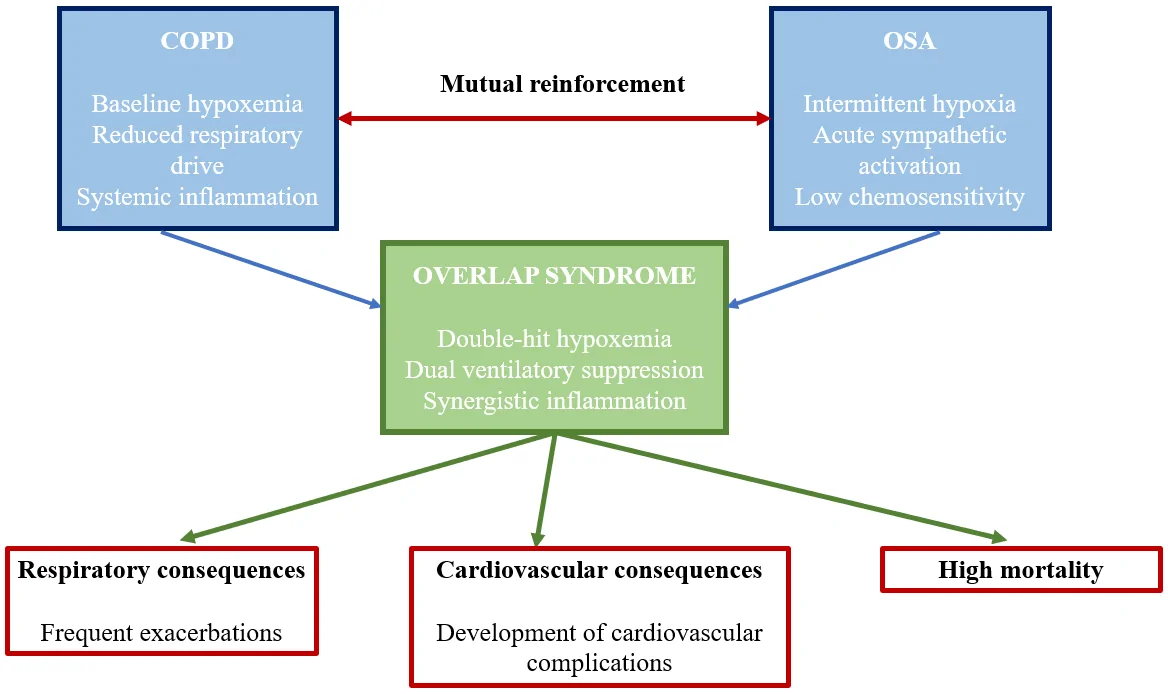
Proposed “double-hit” hypoxemia model: pathophysiological interactions in OVS. COPD, chronic obstructive pulmonary disease; OSA, obstructive sleep apnea; OVS, overlap syndrome.

The degree of nocturnal desaturation is significantly higher in OVS than in either condition evaluated separately, thereby increasing the risk of cardiovascular events [37]. Pulmonary hypertension represents one of the complications where the synergistic mechanism of OVS is most evident: the pulmonary vasoconstriction caused by COPD-related hypoxemia is amplified by the intermittent hypoxic events associated with OSA, leading to a more severe form of pulmonary hypertension, one that is less reversible and harder to treat, compared to the development of this complication in either condition alone. Data from a retrospective comparative study confirmed that patients with OVS face an additional risk of developing pulmonary hypertension (PH), heart failure, and all-cause mortality compared to patients with isolated COPD or OSA (*p* < 0.05 for all parameters) [37]. Atrial fibrillation is a common complication associated with sleep-related respiratory disorders, but its prevalence was higher in patients with OSA [38]. These synergistic interactions are schematically summarized in Figure 1.

## 4. Clinical Phenotypes in OVS

OVS is characterized by clinical heterogeneity, documented in previous observational studies and recognized in the literature, reflecting complex pathophysiological mechanisms. This variability supports the need for a classification system designed to stratify risk and tailor management strategies. The clinical presentation of OVS arises from the interplay between the classic phenotypes of COPD and the characteristics of OSA. COPD is described by two classic clinical phenotypes: the emphysematous type, characterized by low BMI and relatively preserved gas exchange in the early stages, and the chronic bronchitic type, characterized by chronic productive cough, marked hypoxemia, and high BMI, and more frequently associated with right-sided heart failure [39]. Based on data from the literature, we propose a classification of OVS into four main phenotypes, each associated with predominant pathophysiological mechanisms and specific therapeutic implications. The four phenotypes are not mutually exclusive because patients may present with overlapping characteristics. The proposed phenotypic classification should reflect a dominant, clinically relevant pattern that allows for therapeutic intervention, guided by the presence of a representative biomarker, rather than definitively assigning a patient to a category. It represents a guiding framework that can be reassessed as the clinical picture evolves and new clinical and paraclinical details emerge. Table 1 summarizes the source and relative strength of evidence underlying each proposed phenotype. The evidence base is strongest for the hypercapnic phenotype, followed by the emphysematous phenotype, while the obese-metabolic and chronic bronchitic phenotypes rely to a greater extent on extrapolation.

### 4.1. The Obese-Metabolic Phenotype

This phenotype is frequently encountered in OVS and is characterized by the presence of obesity (BMI > 30 kg/m^2^) and a moderate-to-severe form of OSA. Elevated BMI, a known risk factor for upper airway collapse, also serves as an indicator of OSA severity, alongside the apnea–hypopnea index [40]. Furthermore, the presence of cardiovascular disorders, such as right heart failure and peripheral edema, predisposes patients to a rostral fluid shift in the supine position during the night, leading to fluid accumulation in the peripharyngeal tissues [41,42]. A recent retrospective study found that 55.2% of patients with OVS also presented with metabolic syndrome, with an even higher prevalence among those living at altitudes above 2500 m. Compared to those without metabolic syndrome, these patients exhibited more severe nocturnal hypoxemia, indicated by lower mean and minimum oxygen saturation values and prolonged time spent with SpO_2_ < 80%, as well as alterations in hematological and inflammatory profiles. The analysis suggested that the severity of nocturnal hypoxemia is the primary factor underlying the association between OVS and metabolic syndrome, thereby highlighting the role of chronic intermittent hypoxia in the development of diabetes mellitus, arterial hypertension, and pulmonary hypertension. The significance of obesity is also supported by recent interventional data. In the Phase III SURMOUNT-OSA trials, treatment with tirzepatide in patients with moderate-to-severe OSA and obesity resulted in a reduction in respiratory events by 25.3 events/hour in patients not using CPAP and by 29.3 events/hour in those already on CPAP therapy, compared to 5 events/hour in the placebo groups; the estimated differences versus placebo were −20.0 and −23.8 events/hour, respectively (*p* < 0.001 for both trials). The therapy was also associated with significant reductions in body weight, hypoxic burden, and high-sensitivity C-reactive protein (hsCRP) levels, as well as improvements in cardiovascular profiles and symptom severity. Although this study focused on patients with OSA and obesity rather than the OVS population, the results support a therapeutic approach targeting excess adipose tissue, thereby potentially modifying the metabolic, inflammatory, and respiratory mechanisms involved in OVS, and represent a promising strategy for this phenotype, albeit one requiring validation in this specific patient group [43,44].

### 4.2. The Emphysematous Phenotype

The emphysematous phenotype presents a clinical paradox. The destruction of alveolar septa leads to a loss of elastic recoil and the progressive development of hyperinflation, accompanied by air trapping and flattening of the diaphragm. The descent of the diaphragm causes progressive compression of the abdominal organs, thereby promoting early satiety and a progressive reduction in food intake. Driven by increased respiratory effort, the associated energy expenditure, and the inflammatory state, these mechanisms lead to pulmonary cachexia. Thus, the pulmonary hyperinflation associated with emphysema represents a paradox: the increase in lung volume and the caudal traction exerted on the trachea and pharynx reduce the compliance of these structures and alter their susceptibility to collapse during sleep, a mechanism that explains the lower prevalence and severity of sleep apnea syndrome in this phenotype [45]. A study conducted by Krachman et al. involving 51 smokers with moderate-to-severe COPD demonstrated that the degree of emphysema, as assessed by computed tomography, is inversely correlated with the apnea–hypopnea index (AHI) measured via polygraphy: the more severe the emphysema, the lower the AHI, thereby supporting the protective effect exerted on the upper airways [46].

The physiological mechanism underlying this protective effect was evaluated using critical closing pressure (Pcrit), a marker of the pharynx’s tendency to obstruct, with high values indicating an increased risk of collapse and negative values indicating better pharyngeal stability. In patients with COPD and hyperinflation, Biselli et al. demonstrated a significant decrease in Pcrit compared to control subjects, indicating a lower risk of upper airway collapse; furthermore, Pcrit was inversely correlated with functional residual capacity (FRC), with each 1 L increase in FRC associated with a decrease in Pcrit of approximately 1.7 cmH_2_O [20]. These data argue against the hypothesis that the destruction of lung parenchyma and loss of elastic recoil in severe COPD would promote OSA; instead, they confirm the protective effect of hyperinflation on the upper airways. This protective effect is further supported by data regarding the low BMI characteristic of this phenotype, which is associated with lower critical closing pressure and, consequently, better upper airway stability [21,22].

Although hyperinflation may exert beneficial effects regarding upper airway collapse, it does not provide complete protection against the development of obstructive sleep apnea. A study investigating the role of the arousal threshold, a factor actively involved in the pathophysiology of OVS, supports an inverse correlation between this threshold and residual lung volumes (RV and the RV/TLC ratio); this suggests that hyperinflation may alter ventilatory control through mechanisms distinct from those governing upper airway mechanical stability [47]. Indeed, this study highlights the complexity underlying hyperinflation and its role in the development of sleep apnea syndrome, indicating that polysomnographic screening should not be ruled out for patients with this phenotype but rather performed routinely, particularly when highly suggestive symptoms are present.

Functionally, patients present with a reduced membrane diffusion capacity, severe hyperinflation evidenced by increased TLC, and an absent or minimal response to bronchodilators. Nocturnal hypoxemia results from ventilation–perfusion mismatch as well as physiological changes occurring during REM sleep (marked hypoventilation). Therapeutically, the mainstay of treatment for this phenotype is the administration of long-acting bronchodilators, particularly the LAMA/LABA combination [48]. The severity of OVS should not be assessed solely based on the apnea–hypopnea index, as this parameter cannot fully reflect the disease burden in patients who already exhibit impaired baseline gas exchange due to coexisting COPD. Additional parameters of nocturnal hypoxemia—including T90, the oxygen desaturation index (ODI), mean and minimum SpO_2_ values, the duration of individual respiratory episodes, and hypoxic load, provide relevant information beyond the mere frequency of events and should be incorporated into the precision medicine approach proposed in this review [49].

### 4.3. Chronic Bronchitic Phenotype

This phenotype is associated with a higher frequency of emergency department visits due to recurrent exacerbations. The development of apnea episodes may be promoted by mutually reinforcing mechanisms, persistent chronic airway inflammation, mucus hypersecretion, and systemic inflammation, often compounded by an elevated BMI that contributes further through peripharyngeal fat deposition [40]. The clinical picture is dominated by cough, often the initial symptom; mucous expectoration (becoming purulent during exacerbations); and dyspnea quantified using the mMRC scale [50]. Exacerbation severity is significantly increased by the presence of OSA, likely secondary to the amplification of hypoxemia, hypercapnia, systemic inflammation, and ventilatory instability [51].

Chronic hypoxemia triggers compensatory mechanisms with significant clinical consequences. This ventilation–perfusion mismatch leads to hypoxemia and secondary polycythemia, gradually resulting in progressive hypercapnia and respiratory acidosis, phenomena that sustain pulmonary vasoconstriction and, subsequently, the development of chronic cor pulmonale [50]. This secondary polycythemia, an adverse effect of chronic hypoxemia, increases blood viscosity and, consequently, thrombotic risk, representing a complication of OVS. Chronic cor pulmonale is considered the most common adverse outcome in both groups, driven by a much more significant drop in nocturnal oxygen saturation; these patients are also more prone to developing daytime pulmonary hypertension [39,52]. Furthermore, over time, pulmonary hypertension can lead to right ventricular hypertrophy and, eventually, chronic cor pulmonale [50].

From a therapeutic perspective, this hypoxemic–bronchitic phenotype requires a comprehensive approach. The use of APAP therapy may be considered for patients with sleep-related respiratory events that are predominantly obstructive and not associated with nocturnal hypoventilation, hypercapnia, or significant hypoxemia. Given that this phenotype is characterized by chronic hypoxemia and a tendency toward hypercapnia, APAP is generally not the preferred method for this category of patients; BPAP is preferred in the presence of hypercapnia, nocturnal hypoventilation, or significant hypoxemia associated with COPD.

However, PAP therapy complements rather than replaces bronchodilator therapy for COPD, which must be maintained and adjusted according to clinical guidelines [53]. Pharmacological treatment with ICS + LABA + LAMA may be considered for the management of patients with a bronchitic component. However, the initiation of triple therapy must adhere to current guidelines for chronic obstructive pulmonary disease, taking into account the history of exacerbations, blood eosinophil count, response to previous therapies, symptom severity, and response to bronchodilator therapy.

Although these drug classes are associated with increased nocturnal oxygen saturation, they have not demonstrated clear benefits regarding sleep quality [54,55]. The role of macrolides in preventing infectious exacerbations should not be overlooked; a study involving the daily administration of 250 mg of azithromycin for 12 months demonstrated a prolongation of the time to the first exacerbation and a 27% reduction in the risk of exacerbation [56]. As was noted regarding triple therapy, treatment with azithromycin must strictly adhere to the indications established by recognized COPD guidelines concerning chronic macrolide therapy for patients prone to exacerbations, rather than being viewed as specific to the OVS phenotype.

Oxygen therapy (targeting an SpO_2_ of 88–92%) should be considered for patients with OVS who remain hypoxemic despite PAP therapy. However, hypoventilation must be monitored during titration, as BPAP therapy is more appropriate than PAP combined with supplemental oxygen in such cases [57].

### 4.4. The Hypercapnic Phenotype

Chronic carbon dioxide retention and more severe respiratory impairment characterize the hypercapnic phenotype. A study enrolling 163 patients with OVS, 55 with daytime hypercapnia (PaCO_2_ ≥ 45 mmHg) and 108 who were normocapnic, revealed that patients in the former category had a significantly higher BMI (driven by abdominal and neck circumferences) and a greater degree of daytime sleepiness, as assessed via the Epworth Sleepiness Scale. Polysomnographic evaluation showed that these patients experienced much more severe nocturnal hypoxemia, evidenced by the desaturation index, the time spent with oxygen saturation below 90%, and significantly lower mean and minimum oxygen saturation values. Furthermore, hypercapnic patients exhibited greater pulmonary function impairment, with lower FEV_1_ and FVC values, as well as more pronounced daytime hypoxemia. Statistical analysis using logistic regression identified high BMI and reduced FVC as the primary independent determinants of hypercapnia. These findings helped characterize the hypercapnic phenotype of OVS as being defined by obesity, more severe pulmonary function impairment, and a greater degree of nocturnal hypoxemia; this suggests that early recognition of these characteristics can facilitate better risk stratification and the tailoring of therapeutic strategies [58].

It is important to distinguish between the hypercapnic OSA phenotype and coexisting obesity hypoventilation syndrome (OHS), as the latter can contribute significantly to daytime hypercapnia in obese patients with both COPD and OSA. The presence of daytime hypercapnia should not automatically be attributed to the hypercapnic OSA phenotype without ruling out OHS, given the distinct diagnostic criteria and therapeutic management—including a stronger recommendation for non-invasive ventilation (NIV) at higher pressures, depending on the severity of the COPD. If OHS is suspected—for instance, in patients with severe obesity and hypercapnia that is disproportionate to the degree of airflow obstruction—specific evaluation is recommended to quantify the contribution of each condition before attributing the clinical presentation solely to the hypercapnic OSA phenotype.

From a mechanical standpoint, the hypercapnia observed in OVS arises from the combined interaction of the mechanisms underlying both conditions [59]. In COPD, expiratory flow limitation, dynamic hyperinflation, and a significant increase in dead space reduce alveolar ventilation efficiency, thereby promoting CO_2_ retention [60]. Additionally, patients exhibit a blunted ventilatory response to hypoxia and hypercapnia, a trait associated with OSA, which leads to further ventilatory suppression. Consequently, intermittent nocturnal hypoxemia progresses to a persistent and refractory form [61,62], contributing to a clinical phenotype characterized by a more severe course and diagnostic challenges. A distinctive feature is the onset of hypoxemia, hypercapnia, and pulmonary hypertension, even in cases of mild to moderate obstruction, phenomena that are not characteristic of obstructive pulmonary disease in isolation [63].

From a therapeutic perspective, this phenotype requires a non-invasive ventilation approach using BPAP in spontaneous-timed (S/T) mode. While CPAP is effective, in patients with OVS who also exhibit daytime hypercapnia, non-invasive ventilation aimed at reducing carbon dioxide levels is preferable [13]. Furthermore, patients with OVS often experience significant insomnia; however, the use of benzodiazepines and opioids must be avoided due to the risk of central respiratory drive depression, hypercapnia, and an elevated arousal threshold, factors that exacerbate hypoventilation and CO_2_ retention. Consequently, it is safer to prescribe medications that do not belong to these classes when managing insomnia in patients with OVS [64]. Although specific studies on OVS are lacking, data from COPD cohorts demonstrate that benzodiazepine use is associated with an increased risk of acute respiratory failure (aOR 1.56; 95% CI: 1.14–2.13), a risk significantly amplified by concomitant opioid use (aOR 2.32; 95% CI: 1.94–2.77). This is particularly relevant for this phenotype, given the compromised ventilatory reserve [64]. Oxygen supplementation is a key component in treating COPD patients with severe hypoxemia when hypoxemia persists despite optimized therapy; it helps improve oxygenation and reduce mortality without increasing the severity of hypercapnia in these patients [37]. Administration requires careful monitoring in patients with known coronary artery disease or left ventricular dysfunction, as studies have reported an increased frequency of ventricular extrasystoles in patients with COPD and a predisposition to apnea [65,66].

## 5. Proposed Endotypes: A Molecular Framework

The phenotype describes the clinical characteristics of the patient with OVS, whereas the endotype defines the underlying molecular mechanism driving the phenotypic expression. The concept of endotyping, addressed in precision medicine for asthma and, more recently, for COPD in isolation, has not yet been systematically applied to OVS, and the biological heterogeneity of this population remains insufficiently explored [13,15]. Based on available literature and in the absence of a validated, widely accepted classification, we propose a conceptual model comprising three major biological endotypes of the OVS, each characterized by predominant pathophysiological mechanisms, clinically accessible biomarkers, and potential therapeutic implications. Similar to the classification based on phenotypes, this endotypic model represents a synthesis of mechanistic data and indirect biomarkers, rather than a validated diagnostic classification system. A biomarker is a measurable indicator (e.g., blood eosinophils, NLR, or leptin) that may reflect an underlying endotype without independently defining or establishing it. In contrast, a treatable trait is an identifiable, measurable clinical characteristic that can be specifically treated and may stem from a phenotype, endotype, or biomarker, without requiring the demonstration of a fully elucidated biological mechanism. We acknowledge that the level of evidence supporting the proposed endotypes and their mechanistic basis is heterogeneous. The Th2/eosinophilic endotype is supported by a specific inflammatory pathway, whereas the neutrophilic/oxidative and metabolic-adipokine endotypes currently rely predominantly on clusters of associated biomarkers (e.g., NLR, MER, CRP, leptin, and adiponectin) rather than on a clearly established and stable underlying biological mechanism.

This classification is not universal and should not be interpreted as mutually exclusive, as a patient may exhibit manifestations of multiple endotypes simultaneously, but rather serves as a guide for therapeutic decision-making focused on the dominant mechanism. Table 2 provides a synthesis of the sources and the strength of the evidence underlying each proposed endotype. The strongest evidence base supports the neutrophilic/oxidative endotype, while the Th2/eosinophilic and metabolic-adipokine endotypes rely to a greater extent on extrapolations from populations with COPD or OSA, or on mechanistic data/theoretical data.

### 5.1. The Th2/Eosinophilic Endotype

Type 2 (Th2) inflammation is one of the best-characterized immunological mechanisms involved in chronic respiratory diseases. Exposure to external stimuli, such as allergens, microbes, and pollutants, triggers the release of epithelial alarmins (specifically IL-25, IL-33, and TSLP) by airway epithelial cells; this leads to the activation of type 2 helper T (Th2) lymphocytes and type 2 innate lymphoid cells (ILC2), resulting in the subsequent production of cytokines, including IL-4, IL-5, and IL-13 [67,68]. Cytokines derived from the airway epithelium initiate and sustain Th2-type inflammation, leading to eosinophil recruitment, elevated IgE levels, and the development of bronchial hyperreactivity [69,70]. Although Th2 inflammation is characteristic of bronchial asthma, the literature indicates that a similar inflammatory profile is found in 20–40% of patients with COPD; this profile can be identified via peripheral eosinophils, a biomarker of type 2 inflammation, and is associated with a favorable response to inhaled corticosteroids and biological therapies targeting this inflammatory pathway [50,71].

The OVS represents a marked amplification of immune activation and systemic inflammation compared to either pathology evaluated in isolation [61,62]. In patients presenting with a pre-existing or acquired T2 inflammatory profile, this amplification may characterize a distinct endotype, the Th2/eosinophilic endotype of severe asthma, defined by predominant eosinophilic inflammation and specific therapeutic implications, making patients with this inflammatory profile potential candidates for targeted biologic therapy. The eosinophilic endotype in severe asthma is identified using clinically accessible biomarkers (blood eosinophils ≥ 300 cells/μL and/or FeNO > 25 ppb [72]), and the presence of this inflammatory profile opens up prospects for the use of biological therapies to interrupt signaling via the IL-4/IL-13 pathway [73,74].

### 5.2. The Neutrophilic/Oxidative Endotype

The neutrophilic/oxidative profile is frequently encountered in OVS and is characterized by the dominant activation of nuclear factor-κB (NF-κB), triggered by intermittent hypoxia in OSA as well as by chronic exposure to cigarette smoke and other pollutants particularly relevant to COPD, leading to the release of various inflammatory factors [75,76]. Furthermore, a study by Sanchez et al. showed that patients with OVS exhibit higher levels of circulating neutrophils compared to patients with isolated OSA or COPD, suggesting a synergistic effect of the two diseases on systemic neutrophilic inflammation [77]. Additionally, a clinical study demonstrated that hypoxia–reoxygenation cycles generate reactive oxygen species (ROS) in individuals with OSA [78]. These cycles are superimposed on the chronic oxidative stress caused by cigarette smoke in COPD patients; the cumulative effect thus acts upon already compromised airways, amplifying neutrophilic inflammation [79].

However, the consequences of the inflammatory profile in OVS extend beyond the airways: increased platelet activation in OVS patients contributes to the development of atherosclerotic plaques, with lung function playing a significant role in this process [78]. In OVS, hypoxia acts as a trigger for the release of hypoxia-inducible factors (HIF-1α and HIF-2α), mediators generated to facilitate cellular adaptation to low-oxygen conditions, which regulate numerous mechanisms involved in essential adaptive processes. Although considered protective phenomena, their chronic activation leads to a cascade of negative events with implications for pulmonary vasculature [80,81].

The neutrophilic/oxidative profile may characterize a subgroup of patients within the OVS group, characterized by an inflammatory profile and increased cardiovascular risk, requiring a therapeutic approach that differs from that of the other endotypes.

### 5.3. The Metabolic-Adipokine Endotype

This endotype reflects the association between hypoxia, sympathetic activation, and metabolic dysfunction, involving disruption of the adipokine axis (leptin, adiponectin, and resistin), within the context of visceral obesity and insulin resistance. The intermittent hypoxia characteristic of OSA, along with sympathetic activation and oxidative stress, disrupts glucose and lipid homeostasis by activating inflammatory pathways and altering adipokine secretion [82,83].

Furthermore, obesity is associated with increased leptin resistance, a phenomenon implicated in the reduction in the central respiratory stimulus and the development of alveolar hypoventilation. In patients with severe obesity, elevated leptin levels no longer exert a normal stimulatory effect on the medullary respiratory centers; consequently, patients experience diminished neural ventilatory drive and a predisposition to central hypoventilation, a mechanism implicated in the development of refractory hypercapnia in patients with OSA and severe obesity [84].

Adiponectin exerts numerous biological effects, contributing to increased insulin sensitivity in peripheral tissues and exerting anti-inflammatory actions by reducing inflammatory mediators; it also regulates lipid metabolism by promoting fatty acid oxidation and inhibiting hepatic fatty acid synthesis [85]. Adiponectin levels are reduced in the context of the visceral obesity characteristic of the metabolic endotype, and the loss of these protective effects contributes to the amplification of systemic inflammation. It is important to note that there is no linear relationship between adiponectin and COPD: the emphysematous phenotype accompanied by cachexia may paradoxically present with elevated adiponectin levels, reflecting a compensatory response to the patient’s increased catabolism, phenomena that stand in contrast to the metabolic endotype characterized by obesity [86].

The prevalence of metabolic syndrome among individuals with OVS was highlighted in a retrospective study enrolling patients from medium-to-high altitude regions; metabolic syndrome was identified in 55.24% of these patients, and those with both OVS and metabolic syndrome exhibited significantly higher levels of neutrophils, CRP, NLR (neutrophil-to-lymphocyte ratio), and NHR (neutrophil-to-HDL cholesterol ratio), as well as a greater duration of sleep time with oxygen saturation below 80% compared to OVS patients without metabolic syndrome (*p* < 0.05) [87].

This endotype represents an OVS subgroup in which visceral obesity is not merely a comorbidity but a central pathogenic factor that simultaneously influences the severity of OSA and COPD while amplifying cardiovascular risk.

## 6. Biomarkers in OVS: From Endotype to Therapeutic Decision

In OVS, biomarkers should fulfill a dual role: reflecting a dominant pathophysiological mechanism, while remaining accessible, to identify the endotype, and guiding therapeutic decisions. We propose a classification of biomarkers based on the identified endotypes: Th2/eosinophilic, neutrophilic/oxidative, and metabolic-adipokine. This classification is completed by cardiovascular biomarkers, a category that can be assessed across all three endotypes, because the increased cardiovascular risk is a common consequence related to eosinophilic, neutrophilic/oxidative, and metabolic mechanisms, rather than to a distinct endotype.

### 6.1. Biomarkers of the Th2/Eosinophilic Endotype

Blood eosinophil counts can help identify patients more likely to benefit from inhaled corticosteroid treatment and assist in selecting candidates for biologic therapy in cases of concomitant severe eosinophilic asthma [71]. FeNO (fractional exhaled nitric oxide) can guide clinicians in monitoring the response to anti-inflammatory treatment, given that the rate of its decline is directly proportional to the improvement in symptoms [88].

### 6.2. Biomarkers of the Neutrophilic/Oxidative Endotype

Similar to the Th2 endotype, in the neutrophilic/oxidative endotype, a simple peripheral neutrophil count reveals elevated levels in OVS compared to isolated COPD, regardless of CPAP adherence, making it a potentially accessible inflammatory biomarker [89]. Furthermore, the NLR (neutrophil-to-lymphocyte ratio) is easily calculated from the absolute neutrophil and lymphocyte counts obtained from a routine complete blood count. An elevated value reflects the balance between systemic neutrophilic inflammation and the adaptive immune response. Studies have identified an NLR cutoff of ≥2.49 for the presence of OSA in patients already diagnosed with COPD [90]. Another ratio that can be calculated in a similar manner is the MER (monocyte-to-eosinophil ratio); an elevated value reflects the monocytic component of systemic inflammation and a decrease in circulating eosinophils, a pattern observed during COPD exacerbations. Recent studies have demonstrated a significantly higher MER in OVS compared to isolated COPD [91]. It has also been shown that IL-6, hs-CRP (high-sensitivity C-reactive protein), and G-CSF (granulocyte colony-stimulating factor) are elevated in OVS compared to isolated COPD or OSA, suggesting a potential role as biomarkers of systemic inflammatory burden [77]. Although supported by the pathophysiology of chronic hypoxemia in OVS, HIF-1α (hypoxia-inducible factor 1-alpha) has not been sufficiently studied; existing reference data derive from populations with isolated OSA, and no clinically validated threshold has been established [92,93].

### 6.3. Biomarkers of the Metabolic Endotype: Adipokines

Leptin, adiponectin, and resistin have been shown to be significantly altered in OSA patients with a characteristic metabolic profile [94]. An overall altered metabolic profile was observed in OSA patients, with elevated fasting glucose, HbA1c levels, lipid profile, and insulin levels, alongside high erythropoietin (EPO) values [95].

### 6.4. Cardiovascular Biomarkers

Given the cardiovascular consequences of OVS, we propose the use of hs-troponin T and NT-proBNP as biomarkers; levels of these markers are significantly higher in OVS than in isolated OSA, indicating subclinical myocardial injury and cardiac wall stress [96]. Imaging markers of subclinical atherosclerosis, such as carotid intima-media thickness, carotid plaque volume, and arterial stiffness (measured via CAVI [Cardio-Ankle Vascular Index] and baPWV [Brachial-Ankle Pulse Wave Velocity]), have also been found to be elevated in OVS [97,98]. Heart rate variability (HRV) allows for the non-invasive quantification of arrhythmias that are synergistically exacerbated in OVS [99].

### 6.5. Conceptual Framework for Identifying the Predominant Endotype

Routine laboratory investigations, such as complete blood count, hs-CRP, eosinophil count, basic metabolic profile, FeNO, NT-proBNP, and hs-troponin T, are routinely used and available, but their designation as established biomarkers for endotype classification in OVS remains at the exploratory stage. We note that indicators such as NLR and MER, along with the proposed cutoff values, have not been prospectively validated for use in endotype classification in this population and should be considered rather as research tools and not as definitive diagnostic criteria.

Step 1: initial screening. For patients with confirmed OVS, routine investigations are performed at no additional cost: complete blood count (used to calculate NLR and MER), hs-CRP, absolute eosinophil count, and a basic metabolic profile (blood glucose, HbA1c, lipid profile). This allows for the identification of the predominant endotype.

Step 2: investigations targeted at the predominant endotype, in selected patients.

For the eosinophilic endotype (characterized by elevated eosinophils), FeNO is measured, and the initiation of ICS or anti-IL-5 biologic therapy (or other biologics, as appropriate) may be considered.

For the neutrophilic/oxidative endotype, NLR and MER, calculated from a routine complete blood count, can quantify the degree of systemic inflammation. In selected cases, IL-6 and G-CSF may be measured; these are biomarkers with potential utility in assessing systemic inflammation.

Regarding the metabolic endotype, a comprehensive metabolic profile assessment (HbA1c, blood glucose, and lipid profile) is recommended. Where available, leptin, adiponectin, and resistin can provide additional insights into metabolic dysfunction, although their use remains primarily within the realm of research.

For patients in whom cardiovascular involvement is suspected or the echocardiographic assessment is accompanied by abnormalities, regardless of endotype, measurement of NT-proBNP and hs-troponin T is recommended to facilitate the early detection of myocardial stress and subclinical cardiac injury.

**Figure 2:**
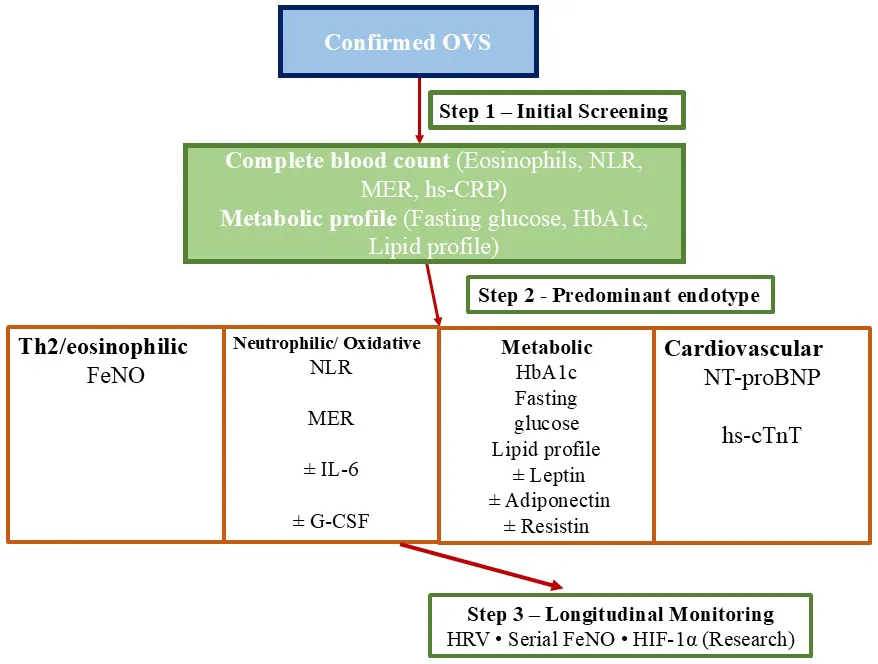
Proposed, non-validated conceptual framework for biomarker-guided management of OVS. Initial screening based on laboratory data, followed by identification of the dominant endotype and selection of specific biomarkers for personalized management and longitudinal monitoring.

Step 3: longitudinal monitoring and the use of emerging biomarkers; HRV, which can be measured using wearable devices, can monitor therapeutic efficacy. Changes in FeNO may help assess the effectiveness of anti-inflammatory therapy. To date, HIF-1α remains exclusively a research tool. Figure 2 illustrates a practical algorithm for the use of biomarkers in OVS, emphasizing the identification of the dominant endotype and the selection of specific biomarkers to support personalized therapeutic decisions.

## 7. Therapeutic Implications

The management of OVS requires a therapeutic approach tailored to the dominant phenotype and the biological endotype identified through diagnostic evaluation. PAP therapy forms the cornerstone of treatment, with evidence showing reduced mortality, driven by fewer hospitalizations and exacerbations, compared to untreated patients [39].

### 7.1. Personalized PAP (CPAP vs. BPAP)

The choice of ventilatory therapy should not be dictated solely by a PaCO_2_ threshold value; instead, it should integrate the severity of sleep apnea, the presence of nocturnal hypoventilation, the potential coexistence of obesity hypoventilation syndrome, the severity of underlying COPD, and evidence of ventilatory failure. CPAP therapy remains appropriate in cases lacking significant daytime hypercapnia or associated nocturnal hypoventilation, whereas BPAP therapy in spontaneous-timed (S/T) mode is preferred for patients with persistent daytime hypercapnia (PaCO_2_ > 45 mmHg)—particularly when nocturnal hypoventilation, severe COPD, or a long-term indication for non-invasive ventilation coexist—because it enables carbon dioxide reduction and aids in the management of ventilatory failure [13,100]. The choice of ventilatory therapy mode must be individualized based on the presence of chronic hypercapnic respiratory failure and sleep-disordered breathing, rather than being guided by a single biochemical threshold value.

Adherence to PAP therapy is an independent predictor of prognosis in OVS; optimal adherence has been associated with a lower risk of all-cause mortality and COPD-related hospitalizations compared to patients with poor adherence [101]. PAP therapy also contributes to reducing systemic pro-inflammatory markers, including CRP and TNF-α, that are implicated in the progression of cardiovascular impairment in OVS, thereby acting on the inflammatory endotype [100].

### 7.2. Dupilumab in the Th2/Eosinophilic Endotype

Dupilumab, a human monoclonal antibody that blocks the IL-4 receptor α subunit (IL-4Rα), a component shared by the IL-4 and IL-13 signaling pathways, is the first biological agent approved for the treatment of COPD with type 2 inflammation, characterized by peripheral eosinophilia ≥ 300 cells/μL [72,102]. In the BOREAS study, dupilumab was directly associated with a reduction in moderate-to-severe annual exacerbations compared to placebo (0.78 vs. 1.10; RR 0.70; 95% CI: 0.58–0.86; *p* < 0.001), alongside significant improvements in FEV1 and quality of life; these results were subsequently confirmed in the NOTUS study (rate 0.86 vs. 1.30 events/year) [102]. Although these studies did not include patients with confirmed OVS, dupilumab’s mechanism of action, blocking the shared pathway independently of the presence of asthma, supports the hypothesis that patients with OVS and a Th2/eosinophilic endotype could benefit from this therapeutic strategy. However, this approach requires studies specifically targeting this population [102].

### 7.3. Incretin-Based Therapies in the Metabolic-Adipokine Endotype

GLP-1 receptor agonists represent one of the most widely discussed therapies for the metabolic-adipokine endotype of OVS, acting simultaneously on excess weight, metabolic dysfunction, and OSA severity. The SURMOUNT-OSA trials, in which tirzepatide was administered for 52 weeks, demonstrated a significant reduction in the apnea–hypopnea index (AHI) compared to placebo, with an estimated difference of −20.0 events/hour in patients not receiving PAP therapy and −23.8 events/hour in those already receiving PAP therapy (*p* < 0.001 for both trials); this was accompanied by significant reductions in body weight, hypoxic burden, hsCRP, and systolic blood pressure [43]. Although the study did not exclusively include patients diagnosed with OVS, the study population, individuals with moderate-to-severe OSA and obesity, overlaps considerably with the metabolic-adipokine endotype of OVS; furthermore, the simultaneous reduction in respiratory events and in inflammatory and metabolic markers suggests that targeting this component may modify the mechanisms underlying the endotype [43,44].

## 8. Discussion

This review proposes a systematic framework for the biological endotyping of the OVS, highlighting the clinical heterogeneity associated with distinct pathological mechanisms and carrying clear implications for therapeutic management. The proposed endotype-based classification has significant limitations that must be acknowledged: endotypes are not mutually exclusive, a patient may simultaneously exhibit characteristics of multiple endotypes, and the classification relies on available observational data rather than on analyses prospectively validated in dedicated OVS cohorts [11,13]. Major gaps exist in the current literature on OVS, including a lack of randomized clinical trials incorporating endotype assessment (given that treatment responses vary significantly by patient subgroup), a lack of consensus regarding the definition and diagnostic criteria for the syndrome, and the exclusion of patients with confirmed OVS from major clinical trials [11].

The proposed phenotypic and endotypic classifications, the “double-hit” model of hypoxemia, and the minimal biomarker panel represent hypotheses derived from indirect evidence and mechanistic extrapolations, rather than externally validated clinical tools. These concepts provide a framework for future research directions and for guiding clinical reasoning, but they require validation through prospective studies before they can be integrated into clinical practice.

Future directions include the early identification of pre-COPD stages and treatable traits, the development of randomized clinical trials to validate therapeutic strategies and guide precision medicine, and the integration of multi-omics data alongside the use of machine learning algorithms for dynamic patient phenotyping [11,15].

Implementing an endotype-based approach to the clinical management of OVS does not necessarily require advanced technologies. A minimal panel of accessible biomarkers, including blood eosinophils, FeNO, daytime PaCO_2_, body mass index, and T90, can facilitate the identification of the dominant endotype and help formulate a targeted therapeutic strategy, thereby contributing to the translation of the precision medicine concept into clinical practice.

It should be noted that the proposed panel has not been validated through prospective studies in the OVS population, and data regarding potential diagnostic performance, such as sensitivity, specificity, predictive value, and OVS-specific reference thresholds, are currently unavailable; therefore, it is proposed as a pragmatic starting point intended to generate hypotheses for clinical reasoning, rather than as a validated diagnostic tool.

## 9. Conclusions

The OVS represents a heterogeneous clinical entity with pathophysiological and prognostic consequences that exceed the sum of the two individual diseases; furthermore, a therapeutic approach focused solely on PAP therapy and bronchodilation is insufficient to address the biological complexity of these patients. This review proposes a structured endotypic framework, comprising Th2/eosinophilic, neutrophilic/oxidative, and metabolic-adipokine endotypes, as a guide for therapeutic strategies targeted at the dominant biological mechanism. Therapeutic options such as dupilumab and GLP-1 receptor agonists offer promising, although not yet OVS-validated, prospects for endotype-guided treatment in OVS, targeting specific inflammatory and metabolic mechanisms relevant to appropriately selected patients with OVS. Although the current literature is based on data from patients with COPD and OSA, it supports the need to evaluate strategies in cohorts consisting strictly of patients with OVS. However, pending the availability of such data, the use of a minimal panel of clinical biomarkers can assist in endotype characterization and guide treatment decisions.

## Figures and Tables

**Table 1:** A summary of the literature sources and the evidence supporting the proposed OVS phenotypes. Depending on the evidence, the resources were classified as directly derived from OVS populations, extrapolated from COPD, OSA, or other conditions studied in isolation, or based on general mechanistic/theoretical data not specific to a clinical population. The strength was derived from the weight of available direct evidence from the OVS population.

Phenotype	OVS Evidence	Extrapolated from COPD	Extrapolated from OSA	Mechanistic/ Theoretical Basis	Strength
Obese-metabolic	-	-	40, 41, 42, 43, 44	-	Limited
Emphysematous	22, 47	20	45, 46	21, 48, 49	Moderate
Chronic bronchitic	51	54, 55, 56	40, 53	39, 50, 52, 57	Limited
Hypercapnic	37, 58, 59, 61	64, 66	-	13, 60, 62, 63, 65	Strong

**Table 2:** Summary of the source and relative strength of evidence supporting the proposed OVS endotypes. Evidence was categorized as directly demonstrated in OVS populations, extrapolated from COPD or OSA/other diseases studied in isolation, or based on general mechanistic/theoretical data not specific to a clinical population. Overall strength was graded qualitatively according to the proportion of direct OVS-specific evidence available.

Endotype	OVS Evidence	Extrapolated from COPD	Extrapolated from OSA	Mechanistic/ Theoretical Basis	Strength
Th2/eosinophilic	61	72	70 [asthma]	50, 62, 67, 68, 69, 71, 73, 74	Limited
Neutrophilic/oxidative	77, 78	-	-	75, 76, 79, 80, 81	Moderate
Metabolic-adipokine	87	86	82, 84	83, 85	Limited

## Data Availability

No new data were created or analyzed in this study. Data sharing is not applicable to this article.
